# Plantar Injuries in Runners: Is There an Association With Weekly Running Volume?

**DOI:** 10.7759/cureus.17537

**Published:** 2021-08-29

**Authors:** Angelo V Vasiliadis, Christos Kazas, Maria Tsatlidou, Polychronis Vazakidis, Dimitrios Metaxiotis

**Affiliations:** 1 2nd Orthopaedic Department, General Hospital of Thessaloniki “Papageorgiou”, Thessaloniki, GRC; 2 Orthopaedic Department, General Hospital of Thessaloniki “Papageorgiou”, Thessaloniki, GRC

**Keywords:** running, foot injuries, stress fractures, tendinopathies, neuropathies

## Abstract

Running is an athletic activity that is increasingly gaining popularity. Despite its benefits, there are many suspected risk factors for running-related overuse injuries. The objective of this study is to describe injuries and clinical symptoms observed on the sole of the foot in runners, giving special attention to the weekly running volume. The literature presented in this narrative review is based on a non-systematic search of the Medline, Google Scholar, and ResearchGate databases and focuses on foot injuries (the full spectrum of the foot pathology from bones to tendons and plantar fascia, nerve, and joint disorders) in runners, which represents an important topic for both professional and recreational runners. The weekly running distance appeared to be one of the strongest predictors for future overuse injuries. Marathon training and average weekly running of over 20 km are possible predictive factors in the development of plantar foot injuries. The plantar medial aspect of the foot is the anatomic area of the foot that most frequently experiences pain, with numerous pathologic conditions. As a result, diagnosis is always a challenging task. The ability to obtain an accurate medical history and carefully perform a physical examination, together with good knowledge of the foot anatomy and kinesiology, are also proven to be key players in ensuring proper diagnosis.

## Introduction and background

One of the most popular sports activities in the adult population around the world is running. As a result, the number of runners and running events has increased steadily since the early 2000s [1]. Running is also an appealing exercise because it is a low-cost, easily accessible form of exercise that offers a number of health benefits [1-2]. Although it has various known health benefits, it is also correlated with an inherent risk for injury. The major negative aspect of running is the high rate of injuries to the lower extremities. Foot injuries are estimated to compose approximately 6% to 40% of all running injuries sustained [3]. The foot is a complex anatomical and biomechanical structure, and, as a result, a careful and thorough medical history and clinical examination are of great importance to confirm a diagnosis and rule out concomitant conditions [4].

Multiple intrinsic and extrinsic risk factors have been outlined that may contribute to plantar injuries, including demographic, biomechanics, anatomic, nutritional, and hormonal factors, as well as training errors and weekly training distance [5]. It is clear that some extrinsic factors, such as improper technique, training surface, and poor footwear, can be avoided [1,3,5]. However, in some cases, the weekly running volume cannot be avoided, particularly in training periods in which athletes are preparing for a half-marathon and/or a marathon [5].

A non-systematic literature search was conducted from 1980 to 2020 on the following databases: Medline, Google Scholar, and ResearchGate. The following search terms were used: "running", "marathon running", "foot injuries", "plantar injuries", "runners", "marathon runners", weekly volume", "weekly kilometers", "stress fracture", "plantar fasciitis", "tendinopathies", "neuropathies", "joint disorders". All retrieved articles were also hand-searched for additional published citations, which were not found through the literature search. Thus, this paper intends to give a compact overview of the background of plantar injuries and provide a systematic guide to the accurate diagnosis of plantar pain in runners. The clinical picture and symptoms of plantar foot injuries are described, and special attention is given to the weekly running volume, which is also briefly discussed. Although the sole of the foot is a small anatomical area, it contains many bones, muscles, tendons, nerves, and other structures. As a result, various types of injuries can occur in almost any of these anatomical structures. Therefore, plantar injuries are the focus of this paper.

## Review

Bone injuries

Metatarsal Stress Fractures

**Figure 1:**
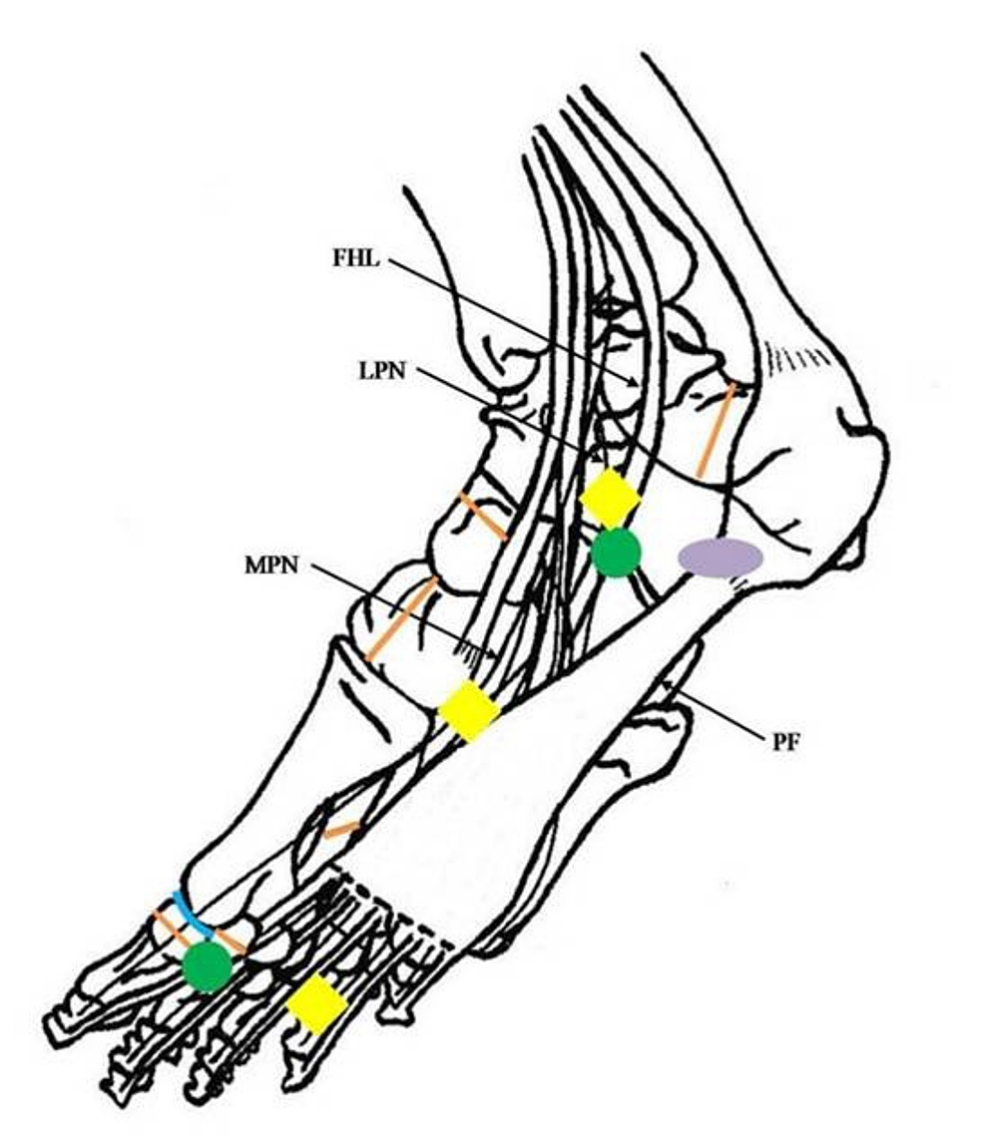
3D anatomical drawing of the plantar aspect of the foot, illustrating the course of the flexor hallucis longus (FHL), the lateral plantar nerve (LPN), the medial plantar nerve (MPN), and the plantar fascia (PF) Colored shapes show the most common and painful anatomical areas and indicate where the pain radiates (orange for bone stress fractures, blue for rigid joints, yellow for nerve injuries, green for tendon injuries, and purple for plantar fasciitis).

Stress fractures in the metatarsal bones are most common in the second or third metatarsals while they are less common in the fourth and fifth metatarsals [5]. The second metatarsal is particularly vulnerable to injury because it is thinner and often longer than the adjacent first metatarsal while it is encompassed more rigidly by the cuneiforms (Figure 1) [3,5]. The fracture typically occurs in the neck or the distal part of the diaphysis with forces being the highest during running. Biomechanical data suggest that foot pronation during running helps distribute the stress throughout the lower extremity and especially in the metatarsal [6]. The overall incidence of metatarsal stress fractures ranges from 10% to 25% of all lower extremity stress fractures [5,7]. The patients complain of forefoot pain, which increases during jogging and running and is relieved during rest. Examination reveals tenderness in the affected bones, inability to toe walk, and sometimes dorsal forefoot swelling [3,8]. A weekly training volume between 90 km and 110 km/week appears to be a crucial factor for the development of a stress fracture at the base of the first metatarsal [5,9].

Navicular Stress Fractures

Navicular stress fractures are considered high risk due to the rate of nonunion caused by poor and unusual vascular supply at the most often affected middle third of the navicular body (Figure 1) [3,10]. Tarsal navicular stress fractures are seen in 1%-3% of all stress fractures [5]. Patients typically present with a history of a vague, aching pain in the dorsal midfoot that may radiate along the medial arch of the foot [3]. In clinical examination, patients often complain of navicular bone pain, particularly at the dorsal aspect or “N spot” (the dorsal aspect of the navicular bone between the anterior tibialis tendon and extensor hallucis longus tendon). Pain may be reproducible with hopping and standing on the toes in the equinus position and typically increases with activity such as running [10]. Anatomical variations, such as flat foot [9], with a recent increase in training volumes, have been reported [10]. This type of injury is found most commonly in track-and-field athletes and in middle-distance runners, but it may be also found in long-distance runners [3,10-11]. Runners who run 64-161 km/week are more likely to present a tarsal navicular stress fracture [5,12-13].

Calcaneus Stress Fractures

Stress fractures of the calcaneus are normally seen in 1.3%-5.7% of all running fractures, followed by metatarsal and tarsal navicular stress fractures [5]. Typically, this injury occurs within the posterior tuberosity (Figure 1), oriented perpendicular to the natural trabecular pattern from dorsoproximal to plantar distal [5]. Common symptoms include plantar or diffuse heel pain with weight-bearing, which is typically relieved during rest. Physical examination reveals pain with medial and lateral compression of the heel. As a result, a positive calcaneal squeeze test with some swelling suggests the diagnosis. Furthermore, there may be pain with palpation of the plantar or posterior heel [8]. A history of an increase in daily training intensity and/or frequency is a risk factor for a possible calcaneus stress fracture [14]. It is notable that a running training program of up to approximately 90 km/week appears to contribute to the development of calcaneus stress fracture [15].

Cuneiform Stress Fractures

 Stress fracture of the cuneiform bones is a rare condition that is a result of torsional and compressive forces between the planted forefoot and the relatively larger hindfoot. Stress fracture of the medial cuneiform is more commonly seen in cuneiform bones (Figure 1) [8]. Unlike bones with a diaphysis, cuneiform stress fractures can be found as transverse sclerotic zone across the long axis of the bone [8]. On clinical examination, significant midfoot swelling and ecchymosis along the plantar foot can be observed. Tenderness is found on the medial aspect of the medial cuneiform bone and the naviculocuneiform joint. Weekly running (25 km/week) is associated with an increased risk of a stress fracture in the medial cuneiform bone [16].

Proximal Phalanx of the Great Toe Stress Fractures

Stress fractures of the foot are common while stress fractures of the toes are rare injuries [17]. Typically, this injury occurs in the proximal phalanx of the great toe, where repetitive higher loads appear during running while the great toe is rapidly flexing upward (Figure 1) [18]. This action often results in an avulsion-type stress fracture of the medial base of the proximal phalanx of the great toe. Physical examination shows mild swelling and pain localized to the medial aspect of the first metatarsophalangeal joint (MTJ). A weekly training volume of 20-42 km/week appears to be crucial for the development of a stress fracture in the proximal phalanx of the great toe [17-18].

Sesamoid Stress Fractures

Stress fractures of the sesamoids are seen less frequently than the above (approximately 1% of all running injuries) [5,19]. The medial sesamoid is more prone to injuries due to the higher loading on it by the first metatarsal head during running (Figure 1) [5]. Pain is typical during the toe-off phase of gait, and findings from physical examination include a restricted and painful range of MTJ motion, tenderness, and diminished plantar flexion [5,20-21]. A high level of running (60-100 km/week) is associated with a significantly increased risk for a sesamoid stress fracture [20].

Plantar Fasciitis

Plantar fasciitis (PF) is one of the most common causes of arch pain and one of the most common foot injuries in runners [11]. PF is usually characterized by localized pain in the plantar fascia insertion in the plantar aspect of the foot, near the medial tubercle of the calcaneus (Figure 1) [2,11]. It seems that the incidence of PF is related to the years of running, the days and kilometers of running per week, and the athlete’s height [11]. Other risk factors for developing PF are pes planus foot structure, excessive pronation, and decreased ankle dorsiflexion [3]. Runners with PF in the acute stage show a lower weekly training volume and a lower loading rate on the hindfoot than those of runners in the chronic stage. This is due to the fact that in the chronic stage of PF, the loss of elasticity of the heel pad leads to failure in the shock-absorbing mechanism, which results in higher loads on the hindfoot [2]. Running has a considerable influence on the occurrence of PF since it is found in 42% of middle-distance runners and 25% of long-distance runners [11]. PF can occur in long-distance runners when their training programs have regimens of 40-130 km/week [2,11,22].

Tendinopathies

Flexor Hallucis Longus Tendinopathy

Tenosynovitis of the flexor hallucis longus (FHL) is an unusual chronic disorder that is associated with long-distance runners who perform repetitive forceful push-offs [4,23]. Pain can occur anywhere throughout the course of the tendon, although the pain is usually localized to the posteromedial ankle [4]. The pain can be elicited with active and passive plantar flexion of the interphalangeal joint of the great toe (Figure 1) [4,23]. A history of moderate swelling and tenderness in the sheath of the FHL behind the medial malleolus and plantar to the sustentaculum tali has been described in a long-distance runner without previous injuries or medical illnesses. Athletes who increase their running weekly schedule (especially from 30 to 50 km/week) are prone to this type of injury [23].

Flexor Hallucis Longus Rupture

Chronic tenosynovitis of the flexor hallucis longus (FHL) tendon can result in complete rupture of the FHL tendon. A potential explanation and possible mechanism of injury is that chronic overuse can lead to thickening of the tendon, ultimately leading to complete rupture [21]. Rupture of the FHL tendon must be diagnosed early because of its potential to lead to a disability. Total rupture of the FHL tendon distal to the knot of Henry can cause loss of push-off strength during walking, jumping, and running [21]. Usually, the FHL tendon ruptures at its fulcrum sites, as it runs around the sustentaculum tali (pain in the medial arch of the foot) (Figure 1) [24-25] and the sesamoid groove (pain in the MTP joint) [26]. A prodromal pain in the push-off phase during running at the plantar aspect of the MTP joint may be revealed from medical history [26]. Acute pain, combined with a popping sensation under the forefoot and accompanied with the loss of the ability to strongly push off from the great toe, is usually described [21,24-26]. Physical examination is likely to reveal mild swelling and tenderness at the plantar aspect of the great toe, no active flexion, and painful passive flexion/extension of the MTP joint of the great toe [26]. Long-distance professional runners [21], a high volume of weekly kilometers (120-150 km/week) [25], and preparation for a marathon are possible risk factors [24,26].

Neuropathies

Morton’s Neuroma

Nerve pathologies are a common cause of lower limb pain in runners [27]. Repetitive traumas to the metatarsals and hyperextension at the MTP joints have been linked to the development of Morton’s neuroma [28]. Runners usually describe neuropathic pain between the third and fourth toes (Figure 1). Burning, cramping, or tingling pain in the toes are common while night pain has been reported [27]. Clinical examination with squeezing inward from the medial and lateral sides of the metatarsals results in pain in the third webspace, which is consistent with Morton’s neuroma. Running four to six times per week and participation in running races (5 to 12 km) are potential risk factors [29].

Jogger’s Foot

Medial plantar nerve entrapment is typically described in runners as a syndrome of neuropathic pain radiating along the medial heel (hindfoot) and longitudinal arch (midfoot) [27-28]. This condition is also known as jogger’s foot [27]. Runners typically report a burning heel pain radiating along the medial longitudinal arch toward the plantar aspect of the first and second toes with accompanying numbness on the sole of the foot behind the great toe (Figure 1) [27-28,30]. Physical examination reveals marked tenderness at the entrapment point of the medial plantar nerve just behind the navicular tubercle while tapping the nerve causes dysesthesia and a positive Tinel’s test. Valgus foot structure, amateur joggers with no previous running profile, and long-distance running (> 25 km) are potential risk factors for medial plantar nerve entrapment [30].

Baxter’s Nerve

Entrapment of the first branch of the lateral plantar nerve (LPN), also known as Baxter's nerve, is commonly encountered in athletes, but it is often misdiagnosed [28]. This syndrome usually causes neuropathic pain in runners, joggers, and ballerinas, so it is important to differentiate heel pain among the athletic population [27-28]. Runners describe pain along the medial heel, usually in the first branch of the LPN as it passes between the abductor hallucis and flexor digitorum brevis and above the quadratus plantae (Figure 1) [28,31]. Clinical examination can reveal tenderness in the proximal plantar fascia and the medial tuberosity of the calcaneus [31] with a positive Tinel’s test [28]. Middle- and long-distance running is a potential risk factor for entrapment of the first branch of the LPN [31-32].

Joint disorders

Hallux Rigidus

Hallux rigidus is a common joint pathology that may contribute to forefoot pain in runners [4,33-34]. During a normal gait, the first MTP joint bears 40% to 60% of bodyweight; however, during jogging and running, the MTP joint bears two to three times and up to eight times bodyweight [34]. Runners may present with pain, crepitus, and stiffness during running [4,33]. The physical examination can reveal pain along the medial border of the great toe (Figure 1) and a limited range of motion in dorsiflexion and plantar flexion. A positive grind test, where the physician applies axial compression and rotation/circumduction motion to the first MTP joint, can be diagnostically helpful [4]. Long-distance runners with a weekly volume of over 40 km/week are at risk of developing hallux rigidus [33].

Table 1 provides the correlation between different types of plantar injuries and the weekly running volumes.

**Table 1:** Plantar foot injuries, anatomic area of pain, and its correlation with weekly running volume PPGT: proximal phalanx of the great toe; NCJ: naviculocuneiform joint; MTP: metatarsophalangeal; FHL: flexor hallucis longus

Category [References]	Diagnosis	Anatomic area of pain	Running (km/week)
Bone injuries [9,12-13,15-18,20]	Metatarsal stress fractures	Forefoot pain, swelling of the dorsum of the foot at the metatarsocuneiform joint	90–110 km/week
	Navicular stress fractures	Dorsal midfoot pain, “N spot” that may radiate to the medial aspect of the longitudinal arch	60–160 km/week
	Calcaneus stress fractures	Plantar heel pain	Up to 90 km/week
	Medial cuneiform stress fracture	Tenderness in the medial aspect of the medial cuneiform bone and the NCJ	Up to 25 km/week
	PPGT stress fracture	Pain around the MTP joint of the great toe	20–42 km/week
	Sesamoid stress fractures	Pain in the left medial forefoot	60–100 km/week
Fascia condition [2,11,22]	Plantar fasciitis	Arch pain, pain in the plantar fascia insertion near the medial tubercle of the calcaneus	Acute: 40 km/week Chronic: 45–130 km/week
Tendinopathies [21,23-26]	FHL tendinopathy	Plantar aspect of MTP joint behind the medial malleolus and plantar to the sustentaculum tali	Increase from 30 to 50 km/week
	FHL rupture	Medial arch of the foot, plantar aspect of the MTP joint, painful passive flexion/extension of MTP joint	120–150 km/week
Neuropathies [29-32]	Morton’s neuroma	Plantar pain between the 3^rd^ and 4^th^metatarsal (3^rd^ webspace)	Races: 5–12 km; Increase weekly running
	Jogger’s foot	Pain in the medial longitudinal arch, burning heel pain	≥25 km/week; Long-distance runners
	Baxter’s neuropathy	Heel pain in the proximal plantar fascia and the medial tuberosity of the calcaneus	Middle/long-distance runners
Joint disorders [33-34]	Hallux rigidus	Pain along the medial border of the great toe, pain with dorsiflexion and plantar flexion of the first MTP joint	≥ 40 km/week

Discussion

Running is one of the most popular and accessible athletic activities among all ages with many beneficial effects, including cardiovascular fitness and skeletal health [1-2]. However, professional and recreational runners are always at risk of injuries to the lower extremities and especially in various structures of the foot. The risk factors that contribute to these injuries have also been reported and divided into non-modifiable and modifiable risk factors [1]. It is assumed that among the modifiable risk factors studied, there is an undoubted correlation between the development of foot injuries and running biomechanics (runner’s technique), as well as the weekly running distance [1,35-36].

**Figure 2:**
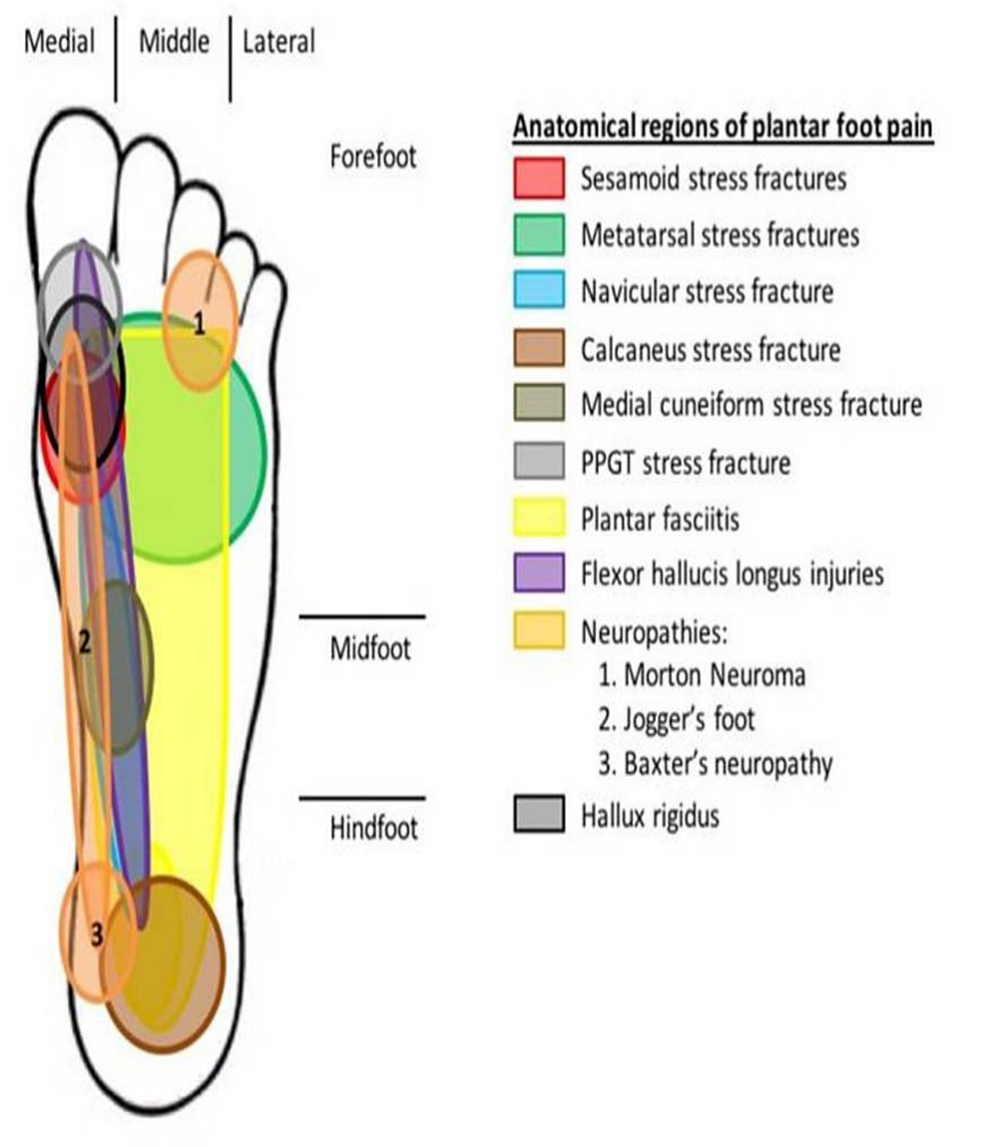
Illustration presents the anatomical regions of plantar foot pain in runners, “the plantar challenge.”

Van Gent et al. [37] conducted a systematic review of injuries in recreational and professional long-distance runners. They reported that the foot is the second most common site of lower extremity running injuries. During running, pronounced forces develop between the foot and the ground while the vertical force approaches more than two times the bodyweight [38]. Wang et al. [39] have suggested that a higher plantar pressure is presented underneath the medial side of the foot during running, despite the running surface. They also reported a long contact time in the medial forefoot when running [39]. These results are identical to those reported by Orendurff et al. [40], who found that there is great peak plantar pressure to the medial forefoot in the anatomic area of the great toe and first metatarsal during the propulsion phase of running. Similarly, in the current study, injuries are commonly described to involve the plantar medial aspect of the foot. The pain that is typically described by athletes radiates from the medial tuberosity of the calcaneus, extends across the medial aspect of the longitudinal arch, and ends at the great toe (Figure 2).

According to the literature, the weekly running distance appears to be the strongest predictor for future overuse injuries [1,35] and may predispose runners to foot overuse injuries affecting bones, tendons, nerves, and joints [3-4]. From the present study, it is clear that the medial column of the foot is prone to injuries (Figure 2). The weekly running distance that can result in an overuse injury in bones is between 20 and 160 km/week [9,12-13,16-18,20]. The respective distance for tendon injuries is 30 to 150 km/week [23-26], that for plantar fascia injuries is 40 to 130 km/week [2,11,22], and that for joint injuries is 40 km/week [33-34]. Nerves are more susceptible to injuries when compared with these anatomical structures (Table 1) [29-30].

**Figure 3:**
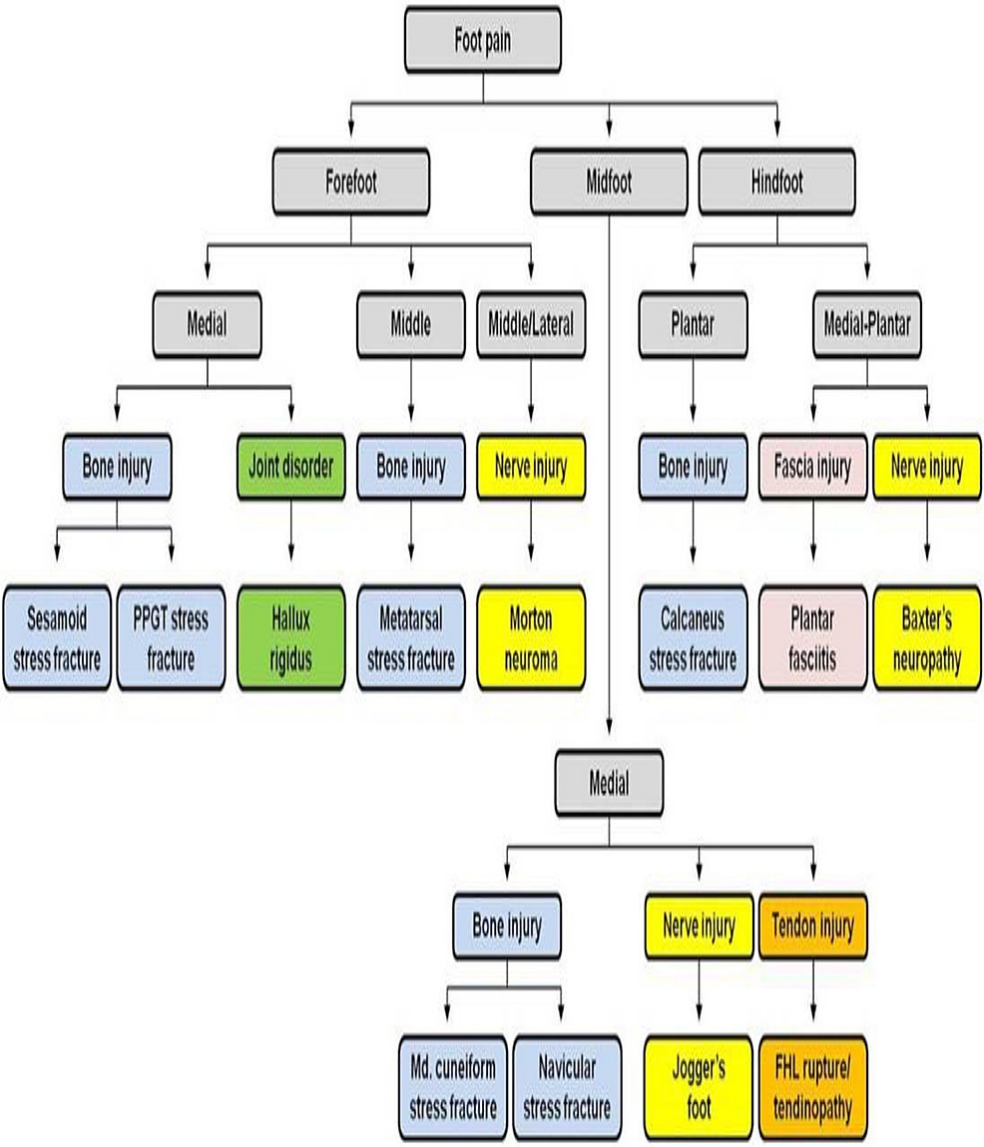
A diagnostic algorithm for plantar foot pain in runners according to the anatomical regions and columns

Anatomically speaking, the foot can be divided into three distinct regions: the forefoot, the midfoot, and the hindfoot (Figure 2). This allows a better step-by-step approach in diagnosing plantar pain in runners. The pain can be localized in a particular anatomical area or/and in a larger area causing a diagnostic dilemma [4,11]. The “plantar challenge” is helpful to physicians but inconvenient for patients [4,11,22]. The diagnostic algorithm described in the current study is based on the findings of the current literature, and its main purpose is to be used as a diagnostic tool for physicians in order to ensure proper diagnosis (Figure 3).

## Conclusions

It is obvious that there is an association between plantar injuries in runners and weekly running volume, and the diagnosis of foot pain in runners can be challenging. Marathon training and average weekly running distances of over 20 km are possible factors in the development of plantar injuries. Overall, a long-distance runner who also competes professionally at a high level are vulnerable to plantar foot injuries. The plantar medial aspect of the foot is the anatomic area of the foot that most frequently experiences pain, with numerous pathologic conditions. As a result, diagnosis is always a challenging task. Generally, a careful medical history and physical examination, together with good knowledge of the anatomy and kinesiology of the foot during running and its relation to the other lower limbs, will in most cases facilitate a more accurate diagnosis and the scheduling of a suitable treatment.
